# Protocol for the Individual Placement and Support (IPS) in Pain Trial: A randomized controlled trial investigating the effectiveness of IPS for patients with chronic pain

**DOI:** 10.1186/s12891-018-1962-5

**Published:** 2018-02-13

**Authors:** Lene Therese B. Linnemørken, Vigdis Sveinsdottir, Thomas Knutzen, Linn Rødevand, Kjersti Helene Hernæs, Silje Endresen Reme

**Affiliations:** 1 Department of Pain Management and Research, Oslo University Hospital, Oslo, Norway; 2 Uni Research Health, Uni Research, Bergen, Norway; 3 Oslo University Hospital, Oslo, Norway; 4 Department of Psychology, Faculty of Social Sciences, University of Oslo, Oslo, Norway; 5 NORMENT, KG Jebsen Centre for Psychosis Research, Division of Mental Health and Addiction, Oslo University Hospital, Oslo, Norway

**Keywords:** Chronic pain, Coping, Individual placement and support, Supported employment, Integrated care, Randomized controlled trial, Vocational rehabilitation, Unemployment, Work disability

## Abstract

**Background:**

Work disability involves large costs to the society as well as to the individual. Work disability is common among people with chronic pain conditions, yet few effective interventions exist. Individual Placement and Support (IPS) is an evidence-based work rehabilitation model originally developed to help people with severe mental illness obtain and maintain employment. The effectiveness of IPS for patients with severe mental illness is well documented, but the model has never before been tested for patients with chronic pain.

**Methods/design:**

The aim of the IPS in Pain trial is to investigate the effectiveness of IPS as an integrated part of the interdisciplinary treatment for patients with chronic pain in a hospital outpatient clinic. The study is a randomized controlled trial comparing pain treatment with integrated IPS to treatment as usual in unemployed patients suffering from various chronic pain conditions. The primary outcome of the study is labor market participation during 12 months after enrollment, and secondary outcomes include physical and mental health and well-being, collected at baseline, 6, and 12 months. Finally, there will be an additional long-term follow-up for the primary outcome, which will be collected through a brief phone interview at 24 months.

**Discussion:**

The IPS in Pain trial will be the first report of the effectiveness of the IPS model of supported employment applied in an outpatient setting for chronic pain patients. It will thus provide important information about the effectiveness of repurposing IPS to a new patient group in great need of job support.

**Trial registration:**

Clinicaltrials.gov: NCT02697656. Registered January 15th, 2016.

## Background

Chronic pain is a major health problem in Norway and elsewhere, with nearly 30% affected in the Norwegian population [1, 2]. Chronic pain often causes significant difficulties in personal and social life, and commonly has negative impact on work ability and participation in the labor market [3]. Absence from the labor market is associated with significant negative individual consequences, such as reduced quality of life and a decline in health [4–7]. Chronic pain also constitutes a substantial socioeconomic burden. In Norway, long-term back pain alone costs more than 4 million euros in sick leave and more than 6 million euros in social welfare annually [8].

**Fig. 1:**
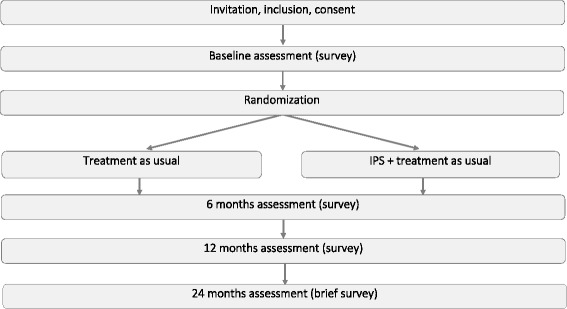
Flowchart

As chronic pain affects many facets of life, it is internationally agreed that complex pain conditions require a multidisciplinary approach [9]. Indeed, evidence suggests that integrated treatment approaches provide better results compared to sequential processes [10]. In integrated approaches, health care professionals from different educational backgrounds assume complementary roles, and their unique expertise is required when the team assesses and treats the patient. The composition of multidisciplinary treatment teams varies, but generally includes three common elements: (1) medical management to reduce symptoms, (2) physical therapy and training to strengthen muscles and improve balance, body awareness and respiration pattern, and (3) cognitive and emotional treatment to enhance coping [9]. We would argue for adding a fourth element: (4) vocational support to manage work. Despite the recent focus on integrating work and health in all patient treatment [11], work-related support is rarely provided in pain clinics (Fig. 1).

Within the field of vocational rehabilitation, it is common to distinguish between two main models; *train and place* and *place and train*. The former is the traditional approach, where training of clients takes place in sheltered workshop jobs, or work experiences in ordinary businesses, with the goal of acquiring necessary skills *before* placement in competitive employment. In Norway, vocational *services as usual* mostly involve train and place approaches, including sheltered training or subsidized work. This is also what pain patients on long-term disability pension are offered from the public welfare services in addition to their medical treatment, with little or no integration between the interventions [12].

The *place and train approach* does not involve prevocational training, but focuses on direct employment in the competitive labor market instead. Individual Placement and Support (IPS) is an evidence-based practice within this approach. The aim of IPS is to provide individual job support to help people with disabilities achieve competitive employment in the ordinary labor market [13]. The model was originally developed for people with severe mental disorders [14, 15], and job support was integrated with mental health treatment rather than being provided as a separate component. A core principle in IPS is that patients are not screened for work readiness, anyone who expresses a desire to work should be supported in order to find a suitable job and environment for that particular individual [16]. Moreover, IPS emphasizes individual preferences and competence. IPS focuses directly on the ordinary labor market, with rapid job search and contact with employers, along with individualized support throughout the process.

Previous studies have consistently shown that IPS produces better work outcomes compared to other types of employment programs for patients with severe mental disorders [17]. In those patients, competitive employment has also been associated with improvements in some non-vocational outcomes, including reductions in outpatient psychiatric treatment and better self-esteem [18]. Despite substantial evidence of the effectiveness of IPS for patients with mental illnesses, the effect of IPS has never been investigated for patients with chronic pain. Pain is an experience that involves affective, motivational, and sensory components [19, 20], and given the similarities and overlap between mental illness and chronic pain [21–24], an investigation of repurposing IPS to chronic pain appears promising. Indeed, in a previous pilot study conducted to inform this trial, participants reported mostly positive experiences with IPS as an integrated part of their interdisciplinary pain rehabilitation [25].

## Methods/design

### Aims and objectives

The main aim of this study is to investigate the effectiveness of IPS as an integrated part of the interdisciplinary treatment for patients with chronic pain in a hospital outpatient clinic. The IPS in Pain trial is designed to answer the following questions:

1. Will IPS result in a higher rate of competitive employment compared to treatment as usual (TAU)?
2. Will IPS result in more total hours of competitive work and higher wages than TAU?
3. Will IPS be more effective than TAU in terms of improving health status and quality of life?
4. Is IPS cost-effective compared to TAU?

### Outcome measures

The primary outcome is the rate of competitive employment during 12 months follow-up after inclusion in the study. Competitive employment rate is here defined as ordinary paid employment in the competitive labor market, using a threshold of at least one day of work, corresponding to the Swedish IPS trial by Bejerholm et al. [26]. Success in employment will also be measured using a range of standardized indicators of employment outcomes commonly used in previous IPS studies [27]. This includes employment duration (e.g. cumulative numbers of weeks worked in all jobs), job intensity (e.g. percentage working at least 20 h a week), and productivity (e.g. total hours worked/wages).

The secondary outcomes concern health status and quality of life, and involve the following questionnaires:

*Health-related quality of life* will be measured using the Euro-Quol Visual Analogue Scale (EQ-VAS), a vertical scale ranging from 0 (“worst imaginable health state”) to 100 (“best imaginable health state”) [28].

*Pain-related disability* will be measured using a modified version of the Oswestry Disability Index (ODI) [29], which consists of 10 items concerning the effect of back pain on different activities of daily life. The modified ODI is identical to ODI with one exception; the word “back” (which occurs once, in the introduction to the form) is deleted. Each item is scored from 0 to 5, with higher values representing more disability.

*Psychological distress* will be measured using the Hopkins Symptom Checklist-25 (HSCL-25) [30]. The questionnaire consists of 25 questions concerning anxiety, depression and somatization. A mean total score of < 1.75 is within the normal range, while a score of 1.75 and above indicates psychological distress in need of treatment.

*Pain intensity and bothersomeness* will be measured using the Numeric Rating Scale (NRS) with scores from 0 to 10. *Pain intensity* will be measured by asking patients to report how intense their pain usually is, from 0 (no pain) to 10 (worst possible pain), and *pain bothersomeness* will be measured by asking how bothersome the pain usually is, from 0 (not bothersome at all) to 10 (worst possible bother).

### Data collection and management

All data will be collected through self-reported surveys. Baseline and follow-up assessments will be collected through a local electronic pain registry already established and in use at the clinic. Each patient assessed at the pain clinic meets one hour prior to their first scheduled consultation to receive a pre-programmed Lenovo tablet with secure software. The tablet has an app (i.e. Infopad) installed for collecting the answers to the study questionnaires. The registry has been approved by the Data Protection Officer at Oslo University Hospital, and requires a dedicated informed consent. The research coordinator or other members of the staff will be available to answer any questions the participants may have during the completion. Paper options will be available.

#### Baseline assessment

All patients complete a standardized screening on the same day as their first consultation at the clinic. Included in the screening are questionnaires about demographical and clinical characteristics, such as physical and mental health and employment status. If the screening was conducted more than two months earlier, the patient will be asked to complete the screening again to ensure reliable baseline data. No procedures related to the trial will take place before the baseline assessment has been completed.

#### Follow-up assessments

The follow-up assessments will be conducted 6 and 12 months after randomization. The research coordinator will perform the follow-up assessments, and the location will either be at the clinic or the participant’s home, depending on their preferences. For the primary outcome (rate of competitive employment) we will also add a long-term follow-up collected through a brief phone interview at 24 months (Fig. 1).

#### Data management

Electronic data collected using tablets will automatically be transferred to and stored in a secure online database. Data collected on paper will be entered manually by the research coordinator, after which the original questionnaires will be destroyed. The information will be de-identified, and the identifier will be secured in a locked and fireproof safe.

### Study design

The study is designed as a randomized controlled trial (RCT) in order to respond to the main aim of the study, which is to conduct an effect evaluation of IPS for patients with chronic pain (Fig. 1). The effect evaluation is further accompanied by a cost-benefit analysis to provide an economic assessment of potential effects of the intervention.

### Participants

#### Number and source of participants

The goal is to recruit 80 participants to the trial (see sample size calculations). Patients will be consecutively recruited and randomized at the Department of Pain Management and Research at Oslo University Hospital, Norway. All patients referred to the clinic eligible for participation will be informed about the study and invited to participate.

#### Recruitment

All clinicians, including the office staff, have been trained in the inclusion procedures and are able to inform and include patients during their consultations. Clinicians can also inform the primary investigator and/or the coordinator of the study about potential participants, who will then contact the patient by phone. Posters and brochures providing information about the study and contact information to the researchers are distributed throughout the clinic, allowing patients to make direct contact if interested.

Patients who want to participate will be informed of their rights according to the Helsinki declaration, receive additional information about the study, and sign a written informed consent before any trial related procedure takes place. The design of the trial will be explained to the patients in detail. For those included, baseline assessment and randomization will be performed.

#### Inclusion criteria

1. Patients referred to the pain clinic and eligible for interdisciplinary treatment
2. Not employed (long-term sick leave, disability pension, or unemployed)
3. Expressed desire to work
4. Age between 18 and 65
5. Living within a reasonable distance to the clinic (i.e. in the city of Oslo)

#### Exclusion criteria

1. Insufficient language skills to answer questionnaires in Norwegian

### Randomization

Participants will be randomly allocated to one of the following conditions: 1) IPS and treatment as usual (the intervention group), or 2) treatment as usual (the control group). The randomization will be conducted through a randomization app called RandomizeIt with a 1:1 randomization ratio. A 2:1 randomization ratio will be applied the first months of recruitment to ensure that the employment specialists obtain sufficient clients in their portfolio. Towards the end of the inclusion period, a 1:2 randomization ratio will be applied to ensure similar group sizes. The principal investigator or the coordinator of the study will inform the participants about the randomization outcome.

### Interventions

Participants randomized to the intervention group will receive job support according to the IPS model from an employment specialist, in addition to treatment as usual. The employment specialists will be included in the interdisciplinary treatment teams at the clinic. In addition to ad hoc meetings on a regular basis, the employment specialists and the pain management team will have monthly meetings where they discuss all participants. The employment specialists will deliver IPS employment support according to a detailed manual [31], and adhering to the eight principles of IPS: (1) eligibility based on the patient’s own choice, (2) focus on competitive employment (i.e., jobs in integrated work settings in the competitive job market at prevailing wages, with supervision provided by personnel employed by the business), (3) integration of mental health and employment services,^1^ (4) attention to patients’ preferences, (5) work incentives planning, (6) rapid job search, (7) systematic job development, and (8) individualized job support [32]. This implies that the job search and support is adapted to the individual’s needs and challenges.

Participants randomized to the control group will receive treatment as usual, which involves interdisciplinary pain management provided by physicians (anesthesiologist, gynecologist, neurologist or specialist in physical medicine and rehabilitation), psychologists, physiotherapists and nurses. Usually, at least two professions follow the patients over a time-period of one to twelve months, with a frequency of every other week to once a month. In addition, participants in the control group will receive a resource manual with information about services and resources for work disabled and unemployed, as well as self-help advice and information about pain management. Finally, those participants who are eligible for vocational rehabilitation provided by the Norwegian Labor and Welfare Administration (NAV)^2^ are advised to contact their local NAV office to receive employment services there.

#### Adaptations of the IPS model to the study context

IPS has traditionally been used for people with severe mental illness. However, the model has also been used for other populations, for example, people with post-traumatic stress disorder, spinal cord injury, traumatic brain injury, and autism spectrum disorder. In the Supported Employment Fidelity Review Manual [33] the authors specifically states that IPS units that serve other populations will connect with another set of service providers than mental health practitioners. Employment services in the current study are thus integrated with interdisciplinary pain treatment and not mental health treatment per se, although the interdisciplinary pain treatment can involve a psychologist.

Furthermore, the organizational model in the current study will include a NAV-coordinator located at the clinic with access to the data systems of all the local NAV offices surrounding the clinic. This position will serve as a supplement to the services provided by the employment specialists, and the position will mainly serve the function of a case manager. Case management in the United States emerged after the deinstitutionalization of patients from the major psychiatric hospitals. A case manager has a coordinating function and is responsible for assessing needs and implementing plans. Although a case manager initially had little direct contact with patients, the modern clinical case manager’s role includes both clinical and social work. In Norway, we do not have an equivalent to a clinical case manager position. Here, the clinical function is placed within the mental health services, and the social function at the local NAV offices. In the current study, we want the NAV-coordinator to serve both these functions. The coordinator will be the point of contact between NAV and both participants and treatment providers, and one of the primary tasks for the coordinator will thus be to give general advice on benefits and work incentives. Since patients referred to the clinic belong to a wide range of different NAV offices, we chose to make this adaptation in order to realistically be able to provide benefits counselling and work incentives planning. Another important task for the coordinator is to provide support to the participants when undergoing important changes in their lives regarding work. The coordinator has training in Motivational Interviewing (MI) and conducts regular meetings with the participants. Most of the meetings take place outside the clinic, often using assertive engagement and outreach in the participants’ community. This part of the coordinator role pertains to the clinical function of the case manager.

### Drop out and non-compliance

Participants who no longer wish to participate in the study can inform the research group of their decision by notifying their employment specialist, treatment provider, or the coordinator of the study. The research coordinator will in any case contact participants who drop out by phone, and ask if they want to report the reason for withdrawal. If reasons are provided, these will be registered on a dedicated drop out form. Whether they only want to withdraw from further employment support, or if they also want to withdraw from future follow-ups, will also be registered. Finally, it will be registered if the participant gives permission for use of data that has already been collected, or if they want these to be deleted. Participants who drop out of the study will still be included in the intention-to-treat (ITT) analyses.

### Analyses

#### Sample size calculations

The sample size calculations are based on input-data from international IPS studies, which have shown a return to competitive employment rate of 23% for the control group and 61% for the IPS group [34]. Considering these rates to be plausible, each group will require 31 participants to reach a statistically significant difference (using a 5% significance level and a strength of 90%). However, as IPS has never been investigated in this patient group before, the effects found in this study may be more moderate than those found in earlier studies conducted with people with severe mental illnesses. A second possible scenario may be a return to competitive work rate of 23% for the control group and 55% for the IPS group, which would require 44 participants in each group. Given that both these scenarios are plausible, we aim to recruit at least 40 participants in each group.

#### Statistical analyses

The analyses will follow the “intention to treat” principle. The randomization will ensure that there are no systematic differences between the IPS and control group at baseline, both when it comes to observable as well as unobservable characteristics. We will compare proportions employed in each group, and investigate whether differences are statistically significant. Additionally, we will perform regression analyses using logistic regression models to investigate predictors of treatment outcome. Groups will also be compared on secondary outcome measures using survey data at baseline and 6 and 12 month follow-up.

#### Health economic analyses

Because IPS is provided in addition to TAU, the intervention is more costly than TAU alone. A health economic analysis will be conducted to investigate the differences in costs relative to differences in effect (e.g. competitive employment rates, hours of work, wage rates, etc.) between the groups. This is commonly referred to as the incremental cost-effectiveness ratio (ICER). The analyses will also include data on health-related quality of life as measured with the EQ-5D, allowing for an estimation of costs per quality-adjusted life-year (QALY).

#### IPS compliance

The IPS fidelity scale is an established measurement to assess adherence to the IPS methodology [35], and fidelity evaluations will be conducted in the initial phase of the project (baseline) and every 6 months until recruitment is completed. The evaluations will be conducted by an independent and experienced evaluator adhering to the well-established Norwegian translation of the IPS fidelity scale [36], and will provide information on competence and methodology in conducting the intervention, as well as the frequency of contact and interaction with the participants. In the Supported Employment Fidelity Review Manual [37] it is recommended that at least two reviewers conduct fidelity reviews to increase reliability of the findings. The recommendations for the interviewing format is conversational rather than a structured interview. There are recommendations for sample questions for each fidelity item, organized by stakeholder groups, although not a complete set of questions to answer each item or subcriteria item. After the review the reviewers independently rate the project on the IPS Fidelity Scale and then conducts consensus scoring to compare ratings, resolve disagreements, and decide upon final ratings. There are, however, several challenges when conducting fidelity reviews this way. One is that reviewer competency can be low, especially in countries where IPS is relatively new, as in Norway, or where the raters are not calibrated with the originators of the model. Another challenge is the unstructured interview form, which makes it difficult to obtain high inter-rater reliability. In addition, the risk of low consistency between both reviews and reviewers due to the individual approach to interview questions and interpretations of answers and observations will always be present.

In the fidelity reviews for the current study we have thus chosen a slightly different approach that involves: 1) a reviewer with operational experience on all levels of IPS, training in doing fidelity reviews from the originators of the model, and calibration with The IPS Employment Center at The Rockville Institute, 2) a fidelity review tool with a matrix of 260 questions specifically developed to answer main items and subcriteria items in the IPS fidelity scale. The 260 questions will be distributed to seven different interview objects, three observational objects and two document objects. All questions are structured with answer options, eliminating the need for interpretation of each question; and 3) a digital scoring tool to avoid errors in scoring data transfer. We believe this will ensure higher reliability as well as consistency across fidelity reviews, with 50% less resources needed.

Any significant deviations from the IPS principles revealed by the fidelity reviews will be noted and communicated back to the employment specialists to ensure and increase quality.

#### Confidentiality

Personal confidentiality is guaranteed for all participants. All data collected in the study is regarded as confidential, and the memory sticks and all data containing personal information will be securely stored in a fireproof safe.

## Conclusion

To our knowledge, the IPS in Pain trial will be the first RCT to investigate the effectiveness of IPS for patients with chronic pain. The study will provide important evidence-based information about the effectiveness of repurposing the IPS model to this new patient group. It will further provide evidence-based knowledge about the value of integrating work rehabilitation in a somatic hospital setting, and the feasibility and effectiveness of providing the job support in a parallel, as opposed to sequential, way.

## Abbreviations

EQ-5D: European Quality of Life – 5 Dimensions
HSCL-25: Hopkins Symptom Checklist - 25
IPS: Individual Placement and Support
ITT: Intention-To-Treat
NAV: Norwegian Labor and Welfare Administration
NRS: Numeric Rating Scale
RCT: Randomized Controlled Trial

## Footnotes

1 See “Adaptations of the IPS model to the study context”

2 NAV administers schemes such as unemployment benefit, work assessment allowance, sickness benefit, pensions, child benefit and cash-for-care benefit.

## References

[1] Rustoen T, Wahl AK, Hanestad BR, Lerdal A, Paul S, Miaskowski C (2004). Prevalence and characteristics of chronic pain in the general Norwegian population. Eur J Pain.

[2] Landmark T, Romundstad P, Dale O, Borchgrevink PC, Kaasa S (2012). Estimatgging the prevalence of chronic pain: validation of recall against longitudinal reporting (the HUNT pain study). Pain.

[3] Overland S, Harvey SB, Knudsen AK, Mykletun A, Hotopf M (2012). Widespread pain and medically certified disability pension in the Hordaland health study. Eur J Pain.

[4] Breivik H, Collett B, Ventafridda V, Cohen R, Gallacher D (2006). Survey of chronic pain in Europe: prevalence, impact on daily life, and treatment. Eur J Pain.

[5] Lame IE, Peters ML, Vlaeyen JW, Kleef M, Patijn J (2005). Quality of life in chronic pain is more associated with beliefs about pain, than with pain intensity. Eur J Pain.

[6] Rueda S, Chambers L, Wilson M, Mustard C, Rourke SB, Bayoumi A, Raboud J, Lavis J (2012). Association of returning to work with better health in working-aged adults: a systematic review. Am J Public Health.

[7] Paul KI, Moser K (2009). Unemployment impairs mental health: meta-analyses. J Vocat Behav.

[8] Ihlebæk C, Lærum E (2004). Plager flest - koster mest - muskel- og skjelettlidelser i Norge.

[9] Schatman ME. Interdisciplinary Chronic Pain Management: International Perspectives. In: Pain Clinical Updates. 2012;20(7). https://s3.amazonaws.com/rdcms-iasp/files/production/public/Content/ContentFolders/Publications2/PainClinicalUpdates/Archives/PCU_20-7_web.pdf.

[10] Lambeek LC, Bosmans JE, Van Royen BJ, Van Tulder MW, Van Mechelen W, Anema JR (2010). Effect of integrated care for sick listed patients with chronic low back pain: economic evaluation alongside a randomised controlled trial. BMJ.

[11] OECD (2013). Mental health and work: Norway.

[12] Nilsen G, Elstad I (2006). Arbeid og verdighet. Pasienters fortellinger fra livet med langvarige smerter. Nordisk Tidsskrift for Helseforskning.

[13] Bond GR, Becker DR, Drake RE, Rapp CA, Meisler N, Lehman AF, Bell MD, Blyler CR (2001). Implementing supported employment as an evidence-based practice. Psychiatr Serv.

[14] Drake RE, McHugo GJ, Bebout RR, Becker DR, Harris M, Bond GR, Quimby E (1999). A randomized clinical trial of supported employment for inner-city patients with severe mental disorders. Arch Gen Psychiatry.

[15] Bond GR (2012). Making the case for IPS supported employment. Adm Paolicy Ment Health.

[16] Corrigan PW (2001). Place-then-train: an alternative service paradigm for persons with psychiatric disabilities. Clin Psychol Sci Pract.

[17] Drake RE, Bond GR, Goldman HH, Hogan MF, Karakus M (2016). Individual placement and support services boost employment for people with serious mental illnesses, but funding is lacking. Health Aff (Millwood).

[18] Luciano A, Bond GR, Drake RE (2014). Does employment alter the course and outcome of schizophrenia and other severe mental illnesses? A systematic review of longitudinal research. Schizophr Res.

[19] Auvray M, Myin E, Spence C (2010). The sensory-discriminative and affective-motivational aspects of pain. Neurosci Biobehav Rev.

[20] International Association for the Study of Pain (2012). IASP taxonomy.

[21] Villano CL, Rosenblum A, Magura S, Fong C, Cleland C, Betzler TF (2007). Prevalence and correlates of posttraumatic stress disorder and chronic severe pain in psychiatric outpatients. J Rehabil Res Dev..

[22] Reme SE, Tangen T, Moe T, Eriksen HR (2011). Prevalence of psychiatric disorders in sick listed chronic low back pain patients. Eur J Pain.

[23] Dersh J, Gatchel RJ, Mayer T, Polatin P, Temple OR (2006). Prevalence of psychiatric disorders in patients with chronic disabling occupational spinal disorders. Spine (Phila Pa 1976).

[24] Von Korff M, Crane P, Lane M, Miglioretti DL, Simon G, Saunders K, Stang P, Brandenburg N, Kessler R (2005). Chronic spinal pain and physical-mental comorbidity in the United States: results from the national comorbidity survey replication. Pain.

[25] Rodevand L, Ljosaa TM, Granan LP, Knutzen T, Jacobsen HB, Reme SE (2017). A pilot study of the individual placement and support model for patients with chronic pain. BMC Musculoskelet Disord.

[26] Bejerholm U, Areberg C, Hofgren C, Sandlund M, Rinaldi M (2015). Individual placement and support in Sweden - a randomized controlled trial. Nord J Psychiatry.

[27] Bond GR, Campbell K, Drake RE (2012). Standardizing measures in four domains of employment outcomes for individual placement and support. Psychiatr Serv.

[28] EuroQol G (1990). EuroQol - a new facility for the measurement of health-related quality of life. Health Policy.

[29] Fairbank JC, Pynsent PB (2000). The Oswestry disability index. Spine (Phila Pa 1976).

[30] Derogatis LR, Lipman RS, Rickels K, Uhlenhuth EH, Covi L (1974). The Hopkins symptom checklist (HSCL). A measure of primary symptom dimensions. Mod Probl Pharmacopsychiatry.

[31] Swanson S, Becker DR (2013). Individual Placement and Support; en praktisk veileder In.

[32] Drake RE, Bond GR, Becker DR (2012). IPS supported employment: an evidence-based approach to supported employment.

[33] Malterud K, Siersma VD, Guassora AD. Sample size in qualitative interview studies: guided by information power. Qual Health Res. 2015; https://www.ncbi.nlm.nih.gov/pubmed/26613970. 10.1177/1049732315617444 26613970

[34] Bond GR, Drake RE, Becker DR (2008). An update on randomized controlled trials of evidence-based supported employment. Psychiatr Rehabil J.

[35] Bond GR, Peterson AE, Becker DR, Drake RE (2012). Validation of the revised individual placement and support Fidelity scale (IPS-25). Psychiatr Serv.

[36] **IPS Fidelity Scale - Norwegian version** [https://ipsworks.org/wp-content/uploads/2017/08/IPS-Fidelity-Scale-Nor-Kvalitetsskalaen-i-IPS-norsk.pdf].

[37] Becker DR, Swanson SJ, Reese SL, Bond GR, McLeman BM: Supported employment Fidelity review manual: a companion guide to the evidence-based IPS supported employment Fidelity scale third edn; 2015.

